# Announcement: IXth International Conference on the Coordination and Organometallic Chemistry of Germanium, Tin, and Lead (ICCOC-GTL-9)

**DOI:** 10.1155/MBD.1997.245

**Published:** 1997


					IXth INTERNATIONAL CONFERENCE ON THE
COORDINATION AND ORGANOMETALLIC CHEMISTRY
OF GERMANIUM, TIN, AND LEAD
Melbourne, Australia: September 20 25th, 1998
Encompassing the following themes:
, synthesis, structure, and reactivity applications in organic
s3mthesis cages and clusters physical methods and theoret/ca/
calculations , reactive intermediates polymers biological
actMties materials chemistry
The program will include a Metals in Medicine Workshop.
CALL FOR PAPERS
Contacts'
Professor Dainis Dakternieks, Chairman, dainis@deakir.edu.au
School of Biological & Chemical Sciences, Deakin University, Gee/ong,
Victoria 3217, Australia
Dr Edward Tiekink, Secretary, etiekink@chemistry.adelaide.edu.au
Department of Chemistry, The University of Adelaide, Australia 5005
Web site
http://www.science.adelaide.edu.au/chemist/conferences/ICCOCGTL/
245

				

